# 5-Fluoro-3-(2-phenyl­hydrazinyl­idene)indolin-2-one

**DOI:** 10.1107/S1600536811015285

**Published:** 2011-05-07

**Authors:** Wen-Bin Wei, Shuo Tian, Hong Shen, Jie Sun, Hai-Bo Wang

**Affiliations:** a College of Food Science and Light Industry, Nanjing University of Technology, Xinmofan Road No. 5 Nanjing, Nanjing 210009, People’s Republic of China; b College of Science, Nanjing University of Technology, Xinmofan Road No. 5 Nanjing, Nanjing 210009, People’s Republic of China

## Abstract

In the title compound, C_14_H_10_FN_3_O, the six- and five-membered rings of the isatin moiety and the six-membered ring of phenyl­hydrazone are nearly planar with r.m.s. deviations of 0.0003, 0.0004 and 0.007 Å, respectively. The dihedral angle between the phenyl ring and the isatin ring system is 6.09 (9)°. The mol­ecular structure is stabilized by a strong intra­molecular N—H⋯O hydrogen bond, leading to the formation of a pseudo-six-membered ring, generating an *S*(6) ring. The crystal structure features inter­molecular N—H⋯O inter­actions.

## Related literature

For the biological activity of isatin derivatives, see: Samus *et al.* (2004 ▶). For bond-length data, see: Allen *et al.* (1987 ▶). For the preparation, see: Vine *et al.* (2007 ▶).
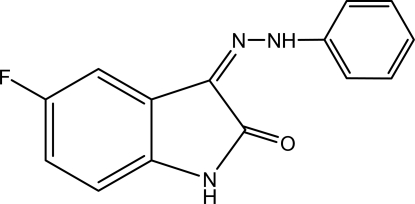


## Experimental

### 

#### Crystal data

C_14_H_10_FN_3_O
*M* *_r_* = 255.25
Triclinic, 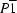

*a* = 6.4840 (13) Å
*b* = 8.7250 (17) Å
*c* = 11.672 (2) Å
α = 69.98 (3)°
β = 75.43 (3)°
γ = 88.33 (3)°
*V* = 599.3 (2) Å^3^
*Z* = 2
Mo *K*α radiation
μ = 0.10 mm^−1^
*T* = 293 K
0.30 × 0.20 × 0.20 mm

#### Data collection

Enraf–Nonius CAD-4 diffractometer
Absorption correction: ψ scan (North *et al.*, 1968 ▶) *T*_min_ = 0.970, *T*_max_ = 0.980
2413 measured reflections
2202 independent reflections
1767 reflections with *I* > 2σ(*I*)
*R*_int_ = 0.014
3 standard reflections every 200 reflections  intensity decay: 1%

#### Refinement

*R*[*F* ^2^ > 2σ(*F* ^2^)] = 0.044
*wR*(*F* ^2^) = 0.153
*S* = 1.00
2202 reflections
173 parameters
H-atom parameters constrained
Δρ_max_ = 0.21 e Å^−3^
Δρ_min_ = −0.20 e Å^−3^

### 

Data collection: *CAD-4 EXPRESS* (Enraf–Nonius, 1989 ▶); cell refinement: *CAD-4 EXPRESS*; data reduction: *CAD-4 EXPRESS*; program(s) used to solve structure: *SHELXS97* (Sheldrick, 2008 ▶); program(s) used to refine structure: *SHELXL97* (Sheldrick, 2008 ▶); molecular graphics: *SHELXTL* (Sheldrick, 2008 ▶); software used to prepare material for publication: *PLATON* (Spek, 2009 ▶).

## Supplementary Material

Crystal structure: contains datablocks global, I. DOI: 10.1107/S1600536811015285/aa2004sup1.cif

Structure factors: contains datablocks I. DOI: 10.1107/S1600536811015285/aa2004Isup2.hkl

Supplementary material file. DOI: 10.1107/S1600536811015285/aa2004Isup3.cml

Additional supplementary materials:  crystallographic information; 3D view; checkCIF report

## Figures and Tables

**Table 1:** Hydrogen-bond geometry (Å, °)

*D*—H⋯*A*	*D*—H	H⋯*A*	*D*⋯*A*	*D*—H⋯*A*
N1—H1*A*⋯O1^i^	0.86	2.05	2.861 (2)	157
N3—H3*A*⋯O1	0.86	2.09	2.760 (2)	135

Symmetry code: (i) 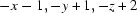.

**Fig. 1.**
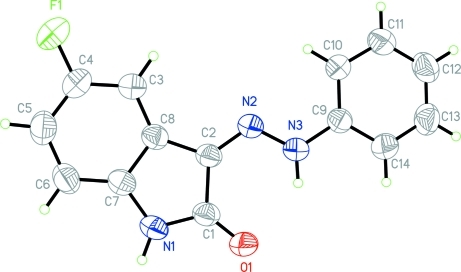
View of the title compound with the atom numbering scheme. Displacement ellipsoids for non-H atoms are drawn at the 50% probability level.

**Fig. 2.**
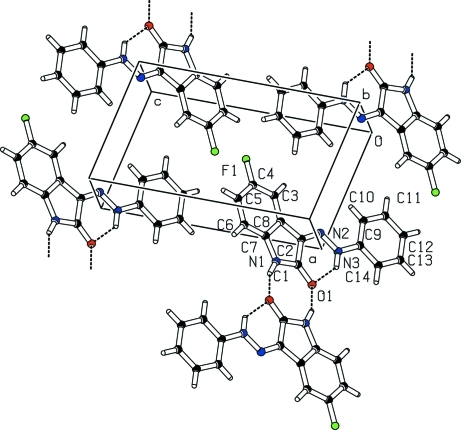
Packing diagram.

## supplementary crystallographic information

### Comment

5-fluoro-3-(2-phenylhydrazono)indolin-2-one is one of isatin derivatives which are reported to show a variety of biological activities such as antibacterial, antimicrobial, antifungal and anti-HIV activities (Samus *et al.*, 2004). We report herein the crystal structure of the title compound.

In the molecule of the title compound (Fig 1), the bond lengths and angles are within normal ranges (Allen *et al.*, 1987). the six- and five-membered rings of the isatin moiety and the six-membered ring of phenylhydrazone are very nearly planar. Rings A (C3—C8) and B(N1/C1/C2/C8/C7) are nearly coplanar, and they are oriented at dihedral angles of A/B = 1.13 (11)°. Rings A (C3—C8) and C(C9—C14) are oriented at dihedral angles of A/C = 6.30 (11)°, and rings B(N1/C1/C2/C8/C7) and C(C9—C14) are oriented at dihedral angles of B/C = 5.90 (11)°.

In the crystal structure, intermolecular N1—H1A···O1 = 2.861 Å interaction may be effective in the stabilization of the structure. The planarity of the molecules was stabilized by a strong intramolecular hydrogen bond N3—H3A···O1 = 2.760 Å leading to the formation of the pseudo six-membered ring(N3/N2/C2/C1/O1/H3A).

### Experimental

For the preparation of the title compound, phenylhydrozone was reacted with equimolar amounts of isatin in ethanol, following the standard procedure (Vine *et al.*, 2007). The product precipitated as yellow crystals in 30 min, and was collected by filtration and washed with cold ethanol. Crystals suitable for X-ray analysis were obtained by slow evaporation of an acetone:ethyl acetate =2:1 solution (yield: 91%, m.p. 543 K).

### Refinement

H atoms were positioned geometrically, with N—H = 0.86 Å (for NH) and C—H = 0.93 Å for aromatic, respectively, and constrained to ride on their parent atoms, with *U*_iso_(H) = *1.2U*_eq_(C,*N*).

### Crystal data

**Table:**

C_14_H_10_FN_3_O	*Z* = 2
*M**_r_* = 255.25	*F*(000) = 264
Triclinic, *P*1	*D*_x_ = 1.415 Mg m^−^^3^
Hall symbol: -P 1	Melting point: 543 K
*a* = 6.4840 (13) Å	Mo *K*α radiation, λ = 0.71073 Å
*b* = 8.7250 (17) Å	Cell parameters from 25 reflections
*c* = 11.672 (2) Å	θ = 10–13°
α = 69.98 (3)°	µ = 0.10 mm^−^^1^
β = 75.43 (3)°	*T* = 293 K
γ = 88.33 (3)°	Block, yellow
*V* = 599.3 (2) Å^3^	0.30 × 0.20 × 0.20 mm

### Data collection

**Table:**

Enraf–Nonius CAD-4 diffractometer	1767 reflections with *I* > 2σ(*I*)
Radiation source: fine-focus sealed tube	*R*_int_ = 0.014
graphite	θ_max_ = 25.4°, θ_min_ = 1.9°
ω/2θ scans	*h* = 0→7
Absorption correction: ψ scan (North *et al.*, 1968)	*k* = −10→10
*T*_min_ = 0.970, *T*_max_ = 0.980	*l* = −13→14
2413 measured reflections	3 standard reflections every 200 reflections
2202 independent reflections	intensity decay: 1%

### Refinement

**Table:**

Refinement on *F*^2^	Secondary atom site location: difference Fourier map
Least-squares matrix: full	Hydrogen site location: inferred from neighbouring sites
*R*[*F*^2^ > 2σ(*F*^2^)] = 0.044	H-atom parameters constrained
*wR*(*F*^2^) = 0.153	*w* = 1/[σ^2^(*F*_o_^2^) + (0.1*P*)^2^ + 0.108*P*] where *P* = (*F*_o_^2^ + 2*F*_c_^2^)/3
*S* = 1.00	(Δ/σ)_max_ < 0.001
2202 reflections	Δρ_max_ = 0.21 e Å^−^^3^
173 parameters	Δρ_min_ = −0.20 e Å^−^^3^
0 restraints	Extinction correction: *SHELXL97* (Sheldrick, 2008), Fc^*^=kFc[1+0.001xFc^2^λ^3^/sin(2θ)]^-1/4^
Primary atom site location: structure-invariant direct methods	Extinction coefficient: 0.058 (11)

### Special details

**Table:**

Geometry. All s.u.'s (except the s.u. in the dihedral angle between two l.s. planes) are estimated using the full covariance matrix. The cell s.u.'s are taken into account individually in the estimation of s.u.'s in distances, angles and torsion angles; correlations between s.u.'s in cell parameters are only used when they are defined by crystal symmetry. An approximate (isotropic) treatment of cell s.u.'s is used for estimating s.u.'s involving l.s. planes.
Refinement. Refinement of *F*^2^ against ALL reflections. The weighted *R*-factor *wR* and goodness of fit *S* are based on *F*^2^, conventional *R*-factors *R* are based on *F*, with *F* set to zero for negative *F*^2^. The threshold expression of *F*^2^ > σ(*F*^2^) is used only for calculating *R*-factors(gt) *etc*. and is not relevant to the choice of reflections for refinement. *R*-factors based on *F*^2^ are statistically about twice as large as those based on *F*, and *R*- factors based on ALL data will be even larger.

### Fractional atomic coordinates and isotropic or equivalent isotropic displacement parameters (Å^2^)

**Table:**

	*x*	*y*	*z*	*U*_iso_*/*U*_eq_	
O1	−0.3760 (2)	0.63511 (17)	1.06116 (14)	0.0569 (4)	
F1	0.5373 (2)	0.7060 (2)	0.55092 (14)	0.0974 (6)	
N1	−0.2569 (2)	0.5813 (2)	0.87432 (15)	0.0520 (4)	
H1A	−0.3724	0.5353	0.8743	0.062*	
C1	−0.2376 (3)	0.6408 (2)	0.96456 (18)	0.0462 (5)	
N2	0.0754 (2)	0.77749 (18)	0.98762 (14)	0.0453 (4)	
C2	−0.0150 (3)	0.7110 (2)	0.92643 (17)	0.0438 (4)	
N3	−0.0413 (2)	0.78890 (19)	1.09465 (15)	0.0494 (4)	
H3A	−0.1751	0.7592	1.1191	0.059*	
C3	0.2929 (3)	0.7207 (2)	0.73265 (18)	0.0539 (5)	
H3B	0.3971	0.7736	0.7502	0.065*	
C4	0.3360 (3)	0.6740 (3)	0.6286 (2)	0.0634 (6)	
C5	0.1875 (4)	0.5940 (3)	0.5986 (2)	0.0671 (6)	
H5A	0.2252	0.5640	0.5273	0.081*	
C6	−0.0170 (4)	0.5592 (3)	0.6761 (2)	0.0587 (5)	
H6A	−0.1202	0.5065	0.6576	0.070*	
C7	−0.0645 (3)	0.6043 (2)	0.78105 (18)	0.0472 (5)	
C8	0.0879 (3)	0.6855 (2)	0.81030 (17)	0.0451 (5)	
C9	0.0503 (3)	0.8486 (2)	1.16853 (17)	0.0451 (5)	
C10	0.2649 (3)	0.8988 (2)	1.13266 (19)	0.0529 (5)	
H10A	0.3526	0.8928	1.0580	0.063*	
C11	0.3471 (4)	0.9576 (3)	1.2088 (2)	0.0632 (6)	
H11A	0.4906	0.9929	1.1841	0.076*	
C12	0.2208 (4)	0.9652 (3)	1.3206 (2)	0.0662 (6)	
H12A	0.2783	1.0050	1.3711	0.079*	
C13	0.0094 (4)	0.9132 (3)	1.3566 (2)	0.0658 (6)	
H13A	−0.0764	0.9169	1.4324	0.079*	
C14	−0.0778 (3)	0.8555 (2)	1.28156 (19)	0.0555 (5)	
H14A	−0.2218	0.8213	1.3066	0.067*	

### Atomic displacement parameters (Å^2^)

**Table:**

	*U*^11^	*U*^22^	*U*^33^	*U*^12^	*U*^13^	*U*^23^
O1	0.0418 (7)	0.0615 (9)	0.0674 (9)	−0.0080 (6)	−0.0031 (6)	−0.0292 (7)
F1	0.0676 (9)	0.1417 (15)	0.0798 (10)	−0.0157 (9)	0.0153 (7)	−0.0571 (10)
N1	0.0429 (9)	0.0550 (9)	0.0631 (10)	−0.0075 (7)	−0.0141 (7)	−0.0255 (8)
C1	0.0401 (9)	0.0430 (9)	0.0556 (11)	−0.0028 (7)	−0.0109 (8)	−0.0176 (8)
N2	0.0416 (8)	0.0456 (9)	0.0487 (9)	−0.0035 (6)	−0.0083 (7)	−0.0182 (7)
C2	0.0390 (9)	0.0426 (9)	0.0498 (10)	−0.0031 (7)	−0.0098 (8)	−0.0167 (8)
N3	0.0402 (8)	0.0559 (9)	0.0520 (9)	−0.0075 (7)	−0.0044 (7)	−0.0230 (7)
C3	0.0469 (10)	0.0590 (12)	0.0554 (11)	−0.0061 (9)	−0.0076 (9)	−0.0225 (9)
C4	0.0560 (12)	0.0751 (14)	0.0538 (12)	−0.0024 (10)	0.0003 (9)	−0.0254 (10)
C5	0.0780 (15)	0.0738 (14)	0.0538 (12)	−0.0002 (12)	−0.0111 (11)	−0.0312 (11)
C6	0.0658 (13)	0.0603 (12)	0.0580 (12)	−0.0040 (10)	−0.0212 (10)	−0.0260 (10)
C7	0.0472 (10)	0.0419 (9)	0.0524 (11)	−0.0011 (8)	−0.0155 (8)	−0.0141 (8)
C8	0.0447 (10)	0.0409 (9)	0.0501 (10)	−0.0006 (7)	−0.0126 (8)	−0.0159 (8)
C9	0.0465 (10)	0.0404 (9)	0.0469 (10)	−0.0015 (7)	−0.0089 (8)	−0.0152 (8)
C10	0.0466 (10)	0.0628 (12)	0.0512 (11)	−0.0037 (9)	−0.0053 (8)	−0.0268 (9)
C11	0.0548 (12)	0.0778 (15)	0.0636 (13)	−0.0071 (10)	−0.0150 (10)	−0.0319 (11)
C12	0.0752 (15)	0.0741 (15)	0.0583 (13)	−0.0067 (12)	−0.0200 (11)	−0.0312 (11)
C13	0.0795 (15)	0.0686 (14)	0.0480 (11)	−0.0029 (11)	−0.0046 (11)	−0.0264 (10)
C14	0.0516 (11)	0.0555 (11)	0.0549 (11)	−0.0047 (9)	−0.0035 (9)	−0.0200 (9)

### Geometric parameters (Å, °)

**Table:**

O1—C1	1.239 (2)	C5—H5A	0.9300
F1—C4	1.363 (2)	C6—C7	1.373 (3)
N1—C1	1.356 (2)	C6—H6A	0.9300
N1—C7	1.401 (2)	C7—C8	1.404 (2)
N1—H1A	0.8600	C9—C10	1.386 (3)
C1—C2	1.483 (2)	C9—C14	1.391 (3)
N2—C2	1.306 (2)	C10—C11	1.380 (3)
N2—N3	1.322 (2)	C10—H10A	0.9300
C2—C8	1.441 (3)	C11—C12	1.377 (3)
N3—C9	1.396 (2)	C11—H11A	0.9300
N3—H3A	0.8600	C12—C13	1.370 (3)
C3—C4	1.371 (3)	C12—H12A	0.9300
C3—C8	1.382 (3)	C13—C14	1.382 (3)
C3—H3B	0.9300	C13—H13A	0.9300
C4—C5	1.382 (3)	C14—H14A	0.9300
C5—C6	1.378 (3)		
			
C1—N1—C7	111.36 (15)	C6—C7—N1	129.42 (18)
C1—N1—H1A	124.3	C6—C7—C8	121.89 (19)
C7—N1—H1A	124.3	N1—C7—C8	108.69 (16)
O1—C1—N1	127.23 (16)	C3—C8—C7	119.75 (18)
O1—C1—C2	126.46 (17)	C3—C8—C2	133.33 (17)
N1—C1—C2	106.29 (16)	C7—C8—C2	106.90 (16)
C2—N2—N3	118.21 (15)	C10—C9—C14	119.59 (18)
N2—C2—C8	125.87 (16)	C10—C9—N3	121.66 (17)
N2—C2—C1	127.33 (17)	C14—C9—N3	118.75 (17)
C8—C2—C1	106.74 (15)	C11—C10—C9	119.34 (19)
N2—N3—C9	120.78 (15)	C11—C10—H10A	120.3
N2—N3—H3A	119.6	C9—C10—H10A	120.3
C9—N3—H3A	119.6	C12—C11—C10	121.3 (2)
C4—C3—C8	117.08 (19)	C12—C11—H11A	119.4
C4—C3—H3B	121.5	C10—C11—H11A	119.4
C8—C3—H3B	121.5	C13—C12—C11	119.2 (2)
F1—C4—C3	118.3 (2)	C13—C12—H12A	120.4
F1—C4—C5	117.90 (19)	C11—C12—H12A	120.4
C3—C4—C5	123.8 (2)	C12—C13—C14	120.8 (2)
C6—C5—C4	119.1 (2)	C12—C13—H13A	119.6
C6—C5—H5A	120.5	C14—C13—H13A	119.6
C4—C5—H5A	120.5	C13—C14—C9	119.79 (19)
C7—C6—C5	118.42 (19)	C13—C14—H14A	120.1
C7—C6—H6A	120.8	C9—C14—H14A	120.1
C5—C6—H6A	120.8		
			
C7—N1—C1—O1	−177.71 (18)	C4—C3—C8—C2	178.50 (19)
C7—N1—C1—C2	0.9 (2)	C6—C7—C8—C3	−0.6 (3)
N3—N2—C2—C8	−178.66 (16)	N1—C7—C8—C3	179.20 (16)
N3—N2—C2—C1	−1.7 (3)	C6—C7—C8—C2	−179.09 (17)
O1—C1—C2—N2	0.7 (3)	N1—C7—C8—C2	0.7 (2)
N1—C1—C2—N2	−177.91 (17)	N2—C2—C8—C3	−0.9 (3)
O1—C1—C2—C8	178.17 (18)	C1—C2—C8—C3	−178.4 (2)
N1—C1—C2—C8	−0.5 (2)	N2—C2—C8—C7	177.35 (17)
C2—N2—N3—C9	175.62 (16)	C1—C2—C8—C7	−0.1 (2)
C8—C3—C4—F1	−179.48 (19)	N2—N3—C9—C10	0.4 (3)
C8—C3—C4—C5	−0.5 (3)	N2—N3—C9—C14	−178.79 (16)
F1—C4—C5—C6	179.6 (2)	C14—C9—C10—C11	−1.2 (3)
C3—C4—C5—C6	0.6 (4)	N3—C9—C10—C11	179.65 (17)
C4—C5—C6—C7	−0.7 (3)	C9—C10—C11—C12	1.0 (3)
C5—C6—C7—N1	−179.05 (19)	C10—C11—C12—C13	−0.1 (4)
C5—C6—C7—C8	0.7 (3)	C11—C12—C13—C14	−0.7 (4)
C1—N1—C7—C6	178.71 (19)	C12—C13—C14—C9	0.5 (3)
C1—N1—C7—C8	−1.0 (2)	C10—C9—C14—C13	0.4 (3)
C4—C3—C8—C7	0.4 (3)	N3—C9—C14—C13	179.65 (17)

### Hydrogen-bond geometry (Å, °)

**Table:**

*D*—H···*A*	*D*—H	H···*A*	*D*···*A*	*D*—H···*A*
N1—H1A···O1^i^	0.86	2.05	2.861 (2)	157
N3—H3A···O1	0.86	2.09	2.760 (2)	135

Symmetry codes: (i) −*x*−1, −*y*+1, −*z*+2.
